# Differences in Muscle Lipogenic Gene Expression, Carcass Traits and Fat Deposition among Three Iberian Pig Strains Finished in Two Different Feeding Systems

**DOI:** 10.3390/ani13071138

**Published:** 2023-03-23

**Authors:** Nicolás Garrido, Mercedes Izquierdo, Francisco I. Hernández-García, Yolanda Núñez, Susana García-Torres, Rita Benítez, José Á. Padilla, Cristina Óvilo

**Affiliations:** 1 CICYTEX-La Orden, 06187 Badajoz, Spain; 2 Departamento de Mejora Genética Animal, INIA-CSIC, Ctra. La Coruña km 7.5, 28040 Madrid, Spain; 3 Facultad de Veterinaria, Universidad de Extremadura, 10003 Cáceres, Spain

**Keywords:** Iberian pig strains, carcass traits, fatty acid composition, gene expression, lipid metabolism, free-range systems

## Abstract

**Simple Summary:**

The Iberian pig is known for the high quality of its products, mostly due to the unique lipid profile of its meat. There are several studies comparing commercial breeds or crosses with the Iberian pig, but there are only a few that compare different strains within the Iberian breed. The aim of this study was to compare carcass traits, fat deposition and gene expression in muscle, of genes involved in lipogenic metabolism in three different strains of Iberian pig finished in two feeding systems. Torbiscal was the strain with the largest lean body development and, therefore, had the highest prime cuts (ham, foreleg, and *L. thoracis*) traits; Retinto strain showed the highest intramuscular fat and MUFA content; while Lampiño was the strain with the highest SFA, PUFA and lipogenic ratios. On the other hand, animals following a concentrate-based diet had greater ham (posterior limb, mainly composed of *semintendinosus*, *gluteus* and *biceps femoris* muscles), *Longissimus thoracis,* prime cuts yields and SFA percentage, than animals following an acorn-based diet. In relation to the gene expression, Retinto and animals following a concentrate-based diet showed the highest expression of several genes related to lipid metabolism. These results evidence the differences between the Iberian strains and the effects of the production system in terms of morphology and lipid metabolism.

**Abstract:**

The Iberian pig breed includes several well-differentiated strains. The present study evaluated carcass traits, fat deposition and muscle expression of important lipogenic genes (*SCD*, *ME1*, *ACACA*, *FASN*, *EGR1*, *ACOX* and *ACLY)* using 65 male pigs of 3 Iberian strains (20 Lampiño, 23 Torbiscal, and 22 Retinto) finished either in a conventional, concentrate-based system (CF) or in *montanera* (MF), a traditional free-range system with acorn feeding. Torbiscal had the highest ham, *Longissimus thoracis* and prime cuts yields, and the thinnest subcutaneous adipose tissue (SAT). Retinto had the highest monounsaturated fatty acids (MUFA) and percentage of intramuscular fat (IMF), while Lampiño had the greatest content of saturated fatty acids (SFA), polyunsaturated fatty acids (PUFA), atherogenic (AI) and thrombogenic (TI) indexes in SAT. Conventionally finished pigs had the highest ham, *L. thoracis* and prime cuts yields, and SFA. *Montanera*-finished animals had the highest PUFA and MUFA contents, and the lowest AI, TI and n6/n3 ratio in SAT. In relation to gene expression, Retinto had the greatest *SCD*, *FASN* and *ACLY* levels. Most studied genes were overexpressed in CF pigs. In conclusion, MF pigs had healthier fat than CF pigs, and Retinto had the healthiest fat and the greatest lipogenic trend in muscle, supported by IMF and lipogenic gene expression.

## 1. Introduction

The Iberian pig is characterised by a large amount of subcutaneous and intramuscular fat with a high content of MUFA and, therefore, the meat products have excellent organoleptic and healthy attributes [1,2]. Their characteristic fat metabolism is mainly due to their genetics [3] and to the particular feeding and rearing system [4]. The Iberian meat production is marketed following a specific regulation “*Norma de Calidad del Cerdo Ibérico*” [5] that describes the age and weight requirements according to the genotype and feeding and rearing system during fattening. This regulation defines two genotypes (pure Iberian and Duroc x Iberian) and three types of finishing systems: intensive, extensive and *montanera* [6]. *Montanera* is a free-range finishing system typical of the southwestern Iberian Peninsula where Iberian pigs are fed on acorns (mainly from *Quercus rotundifolia* Holm oak) and pastures in the *dehesa* (sparsely forested grasslands derived from the Mediterranean forest). During the finishing period, feeding changes progressively from the pre-finishing restricted level (around 1.8 kg/head/day) to *ad libitum* (usually 5–6 kg/head/day of concentrate, or more if acorn-fed), and feed composition changes from an energy/protein balanced concentrate to a high-energy diet based on concentrate (regular extensive system) or on acorn and pastures (*montanera* extensive system). Acorns have high carbohydrate and low protein content, and are richer in MUFA than conventional finishing diets [7,8].

Within the Iberian breed, there are five different recognised strains that show an important degree of variability [9], with Retinto (R), Torbiscal (T) and Lampiño (L) being the main ones. The carcass and meat performances of these Iberian strains have been compared by different authors with different approaches and results. On one hand, Benito et al. (2000) [10] compared only R with T strains and found a tendency towards a greater percentage of intramuscular fat (IMF) in the R strain, and also possible effects of heterosis. Years later, Ibáñez-Escriche et al. (2014) [11] compared these two strains and their crosses and determined that a T x R crossbreed improved the growth and slaughter carcass traits. Later, González et al. (2018) [12] performed the same comparison and determined that R had a higher intramuscular fat deposition than T, confirming the results of Benito et al. (2000) [10]. On the other hand, Muriel et al. (2004) [13] and Clemente et al. (2008) [14] studied T, R and L strains at the same time, with similar results: the former stated that T strain had the lowest IMF of all three strains, while Clemente et al. reported that L strain had the highest IMF—reports that were in line with the findings of Astiz and Alfranca (1998) [15]. Recently, Izquierdo et al. (2018) [16] found that the T strain had a thicker outer SAT layer of subcutaneous fat tissue than the R and L strains. In relation to the feeding system, Tejeda et al. (2002) [17] and Cava et al. (1997) [18] reported a greater IMF in pigs finished in the *montanera* system than in those finished with a concentrate-based diet.

Fatty acid profiles may change according to both the feeding system and the genotype. For example, Muriel et al. (2004) [13] reported no significant differences in fatty acid composition among Iberian strains fed in the *montanera* system. Additionally, Ovilo et al. (2014) [19] concluded that a MUFA-rich diet resulted in a decrease in the SFA content and in an increase in the ratio of MUFA to PUFA. Similarly, pigs fed acorns had larger proportions of MUFA and PUFA and a smaller n6/n3 index than pigs fed a concentrate-based diet [20,21,22].

Few studies have evaluated the expression of genes involved in lipid metabolism in relation to the Iberian pig strains in their different finishing systems. The *SCD* (stearoyl-CoA desaturase) gene, responsible for the biosynthesis of MUFA, was upregulated in Iberian pigs when compared with pure Duroc pigs [22], and also when compared with the Duroc x Iberian F1 genotype [23]. Moreover, when comparing Iberian R and T strains and their diallelic crosses, Villaplana-Velasco et al. (2021) [24] found that the R genotypes had the highest content of PUFA and SFA because the genes related to fatty acid catabolism were downregulated in this genotype.

To delve into the above-described findings, the aim of this study was to evaluate both the differences in carcass and fat deposition and the differences in the muscle expression of genes regulating lipid metabolism in the three main Iberian pig strains fed in two different extensive systems, i.e., conventional (concentrate-based) versus *montanera*.

## 2. Materials and Methods

### 2.1. Animals and Tissue Sampling

All animal procedures were conducted according to the regulations of the Spanish Policy for Animal Protection (RD53/2013), which meets the European Union Directive 2010/63/EU regarding the protection of animals used in research. Animal research protocols were assessed and approved by the Ethics Committee of the University of Extremadura, Spain, ref number 152/2020. The animals of this study, performed in a commercial farm, were raised and slaughtered according to the Spanish and EU legislation on pig production, and all samples were taken post-mortem.

Sixty-five male Iberian pigs of three different strains (20 L, 23 T and 22 R) were randomly selected from the same farrowing group. All animals were vaccinated following the standard protocol of the commercial farms in the region (for swine erysipelas and Aujeszky’s disease). After weaning, all pigs were raised in specific facilities with large outdoor corrals and were fed the same commercial concentrate during the growing period, until they reached an average of 90–100 kg of live weight and then were submitted to finishing, which corresponded to standard Iberian pig raising procedures. At the start of the finishing period (mean body weight 103.85 kg), pigs were separated into 2 groups by randomization in a balanced way according to genotype: 31 pigs (9 L, 11 T and 11 R) were finished with a conventional, balanced concentrate diet (concentrate finishing: CF; Supplementary Table S1) and 34 pigs (11 L, 12 T and 11 R) were finished in a traditional *montanera* system (*montanera* finishing: MF) with a diet based on acorns and pasture in a free-range system. These two finishing systems correspond to Iberian pig official standard production systems (*Norma de Calidad*; [5]). After the finishing period (which lasted for around 12 weeks), pigs were slaughtered at an average weight of 155, 164 and 169 kg and an age of 490, 475 and 500 days, for the L, R and T genotypes, respectively. *L. thoracis* samples at the 10th rib level were obtained at the slaughterhouse, immediately frozen in liquid nitrogen, and then stored at −80 °C until gene expression analysis. Additionally, *L. thoracis* samples were taken for determination of IMF, and samples of subcutaneous fat tissue were collected from the dorsal region of the lumbar area for fatty acid (FA) composition analysis following official regulation [5].

### 2.2. Carcass Traits

Before slaughtering, animals were weighed, and ultrasound scans were performed at the 10th rib level (between the 10th and 11th ribs) with a scanner machine (ALOKA-500SSD, ALOKA Inc., Tokyo, Japan) provided with a 12-cm-long linear probe. Ultrasound images were digitalised in a computer and measured, using Image-J software (U.S.A.), specifically the thickness of the 3 SAT layers: inner subcutaneous adipose tissue (ISAT), middle subcutaneous adipose tissue (MSAT) and outer subcutaneous adipose tissue (OSAT). Total subcutaneous adipose tissue (TSAT) was also measured. At the packing plant, carcass and prime cuts (ham, foreleg, and *L. thoracis*) were obtained and weighed. Prime cut yields were calculated, namely, ham yield (HY), foreleg yield (FY), *L. thoracis* yield (LY), and total prime cuts’ yield (TPCY).

### 2.3. Intramuscular Fat Content Determination

To calculate IMF, fat was extracted from the meat by using chloroform:methanol according to the methodology of Folch et al. (1957) [25]. Fatty acid composition was calculated by increasing the polarity of aminopropil columns to separate the different lipidic fractions according to the methodology of García-Regueiro et al. (1995) [26]. Fatty acid quantification was performed by using gas chromatography according to the methodology of Cava et al. (1997) [18].

### 2.4. RNA Extraction, Quality, and Integrity

The *Ribopure* kit was used for RNA extraction from *L. thoracis* samples. A small portion of sample (90–100 mg) was employed, using the standard protocol. To check the RNA quality, a Nanodrop spectrophotometric device (NanoDrop Technologies, Wilmington, DE, USA) was used.

### 2.5. RNA Retrotranscription

To analyse gene expression, RNA was converted into cDNA through a retrotranscription (RT) procedure using the Superscript II protocol (Invitrogen, Life Technologies, Paisley, U.K.) and random hexamers following the kit’s instructions. To check that the RT was successfully accomplished, standard PCR was performed, followed by an electrophoresis. Then, efficiency and stability tests were performed both in the studied and endogenous genes (*TBP*, *B2M*). Thereafter, quantitative-PCR (qPCR) was assessed using the LightCycler 480 SYBR Green I Master protocol of Roche (Roche Diagnostics Gmbh, Mannheim, Germany). Data obtained from the qPCR were normalised with genorm software using the previously selected endogenous genes.

### 2.6. Genes Studied in the Expression Analyses

Several genes related to lipogenesis were selected for this study because of their relevance in the metabolism of lipids and FA that directly influence the quality of Iberian pork products. These genes are described in Table 1 and include *SCD*, *ME1*, *ACACA*, *FASN*, *EGR1*, *ACOX1* and *ACLY*. The primers employed for the amplification of all the studied candidate genes and of *TBP* and *B2M* as the endogenous genes are shown in the Table 2.

### 2.7. Statistical Analysis

The statistical models corresponded to a factorial design of three strains and two finishing systems. All samples were collected post-mortem in every animal, the animal being the experimental unit. Data from carcass traits, fatty acid composition and normalised gene expression were analysed with the GLM procedure, including strain, finishing system and the interaction, as independent variables. *p*-values below 0.05 were considered significant and those below 0.10 were considered as trends. Least square means of each effect, model mean square error, and *p*-Values are presented in all tables. The GLM procedure was applied using Version 9.3 of the SAS software (SAS Institute Inc., Cary, NC, USA).

## 3. Results

### 3.1. Carcass Traits

Carcass and related traits are depicted in Table 3. The three Iberian strains had different mean slaughter body weights of 155.2, 169.5 and 166 Kg for L, T and R (*p*-Value of <0.0001). There were significant differences in prime cuts yields among genotypes. Torbiscal had greater ham, *L. thoracis*, and total prime cut yields than R, and similar ham yield to L. Retinto and L prime cut yields were similar, except for foreleg yield, which was greater for L than for R. In relation to the feeding system, pigs of the three genotypes fed concentrate had greater prime cut yields than those fed acorns in the *montanera* system.

### 3.2. Fat Deposition

Retinto had the greatest IMF, although this finding did not reach the statistical significance threshold (*p* < 0.079) as shown in Table 3. Additionally, R had significantly thicker TSAT and ISAT than Torbiscal. Lampiño had intermediate SAT thickness values, which were not significantly different to those of T and R, but T had the thickest OSAT (*p* < 0.05). *Montanera*-finished pigs had the thickest TSAT and ISAT, with no difference for the OSAT between finishing systems. This suggests that the acorn diet (characterised by a high-carbohydrate and a low-protein content in relation to commercial diets) increases subcutaneous adipose tissue deposition reducing muscle accretion. The interaction effect (strain x finishing system) was only significant for the MSAT and OSAT thicknesses (*p* = 0.05 and *p* = 0.017, respectively). For these traits, the difference between MF and CF was only significant within T, and there were no significant differences in the other two strains.

### 3.3. Fatty Acid Composition

The results corresponding to these traits are depicted in Table 4. Lampiño had the largest percentage of C16:0, C17:0, C17:1, C18:0, C18:2, SFA and PUFA, the largest atherogenic (AI) and thrombogenic (TI) indexes, and the lowest percentage of C20:0, C18:1 and MUFA. Retinto, on the other hand, had the highest percentages of C18:1 and MUFA, and the lowest percentage of SFA and n6/n3 ratio. Torbiscal, in turn, showed the greatest C20:1 content and the smallest C14:0, C16:1 and C18:3 contents.

In relation to the finishing systems, MF pigs had significantly larger proportions of C18:1, C18:2, C18:3, MUFA and PUFA than CF animals. In contrast, CF pigs deposited more SFA than MF pigs, except for C12:0. Consequently, the proportion of SFA and the AI, TI and n6/n3 indexes were greater in CF pigs than in MF pigs.

Significant interaction effects between genotype and feeding system were found for several fatty acids. For C17:0 and C17:1 deposition, there was a quantitative interaction, both being greater for pigs in the CF than for those in MF, with the L strain showing the largest difference between feeding systems. There was another interaction for the C18:2 fatty acid (*p* = 0.047); this fatty acid deposition was greater in the MF than in the CF for the Lampiño strain. The n6:n3 ratio also had a genotype x system interaction (*p* = 0.031); in this case, the MF animals had no significant differences among strains, while in the CF animals, the R strain had significantly lower values of n6/n3 than the other two strains.

### 3.4. Gene Expression

Table 5 depicts the mean values of the normalised relative expression data obtained by qPCR for the studied genes (*SCD*, *ME1*, *ACACA*, *FASN*, *EGR1*, *ACOX1* and *ACLY*) in the three strains and two finishing systems. The genotype affected the expression of *SCD*, *FASN*, *ACLY* and *ME1* genes. There was a marked, significant genotype effect on the *SCD* and *FASN* gene expressions, which were greater (around 2–3 fold) in R than in the other genotypes. Additionally, *ACLY* showed the same differential expression pattern, being overexpressed in R but with a lower expression difference between genotypes (fold changes 1.3–1.4). The *ME1* gene expression was significantly greater for the L than for the T genotype, with R having intermediate expression values. No significant differences were found for *ACACA*, *EGR1* and *ACOX1* gene expressions among genotypes.

In relation to the feeding system effect, all genes, except for *SCD* and *EGR1*, were significantly overexpressed in CF pigs. There were also interactions between genotype and feeding system for the expression of three genes: *ACACA* (*p* > 0.004), *FASN* (*p* > 0.03) and *ACLY* (*p* > 0.0001). All these interactions were caused by a significant overexpression of those genes in the CF group only for the R strain, while there were no significant differences in the other two strains between CF and MF.

**Figure 1:**
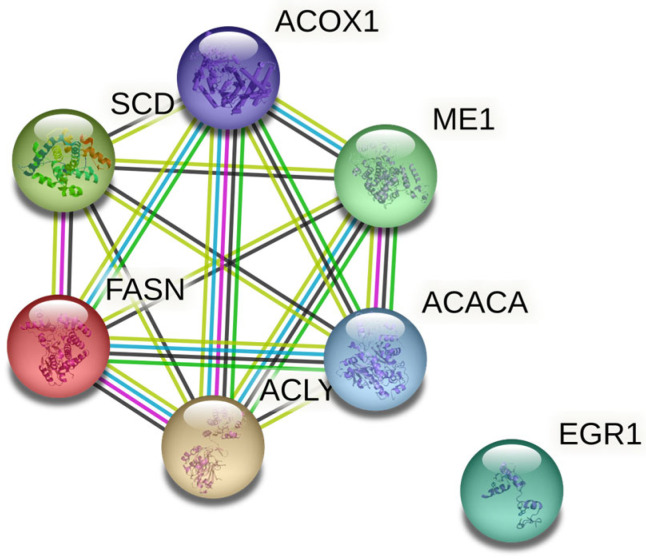
String-db interaction map of all genes involved in this study, network nodes represent proteins and edges represent protein–protein associations that are meant to be specific and meaningful (blue: known interactions from curated databases; magenta: known interactions experimentally determined; green: predicted interactions; black: co-expression; light green: text mining).

The functional relations among these genes were analysed with the string-db tool, and are presented as Figure 1. According to the resulting network, all genes except for *EGR1* were functionally related.

## 4. Discussion

Several studies have found differences in fat deposition and lipid metabolism between modern (industrial, conventional) and local traditional pig breeds [36,37,38]. In addition, Tejeda et al. (2002) [17] concluded that traditional pigs tend to deposit more fat and less muscle than the industrial ones which have been selected for lean meat production.

Clemente et al. (2006) and Dieguez (2000) [39,40] studied the genetic origin of the Iberian strains, and several authors [14,15,16,41] compared fat deposition and lipid metabolism among different strains within the Iberian breed, with some differences in their findings that were not fully coherent. Additionally, differences in the expression of genes related to lipid metabolism have previously been studied comparing pure Iberian vs. Duroc x Iberian crossbreeds [23], pure Iberian vs. pure Duroc [22], and among Iberian strains and their crossbreeds [24]. In the present study, not only genotype, but also feeding system significantly affected fat deposition and fatty acid profiles, as also reported by Daza et al. (2007), Martins et al. (2015), and Rey et al. (2006) [4,21,42], as well as the expression of relevant genes involved in muscle lipogenic processes.

### 4.1. Fat Deposition and Muscle Accretion

Regarding the genotype effect, the T strain had greater main cuts yields than R, being L intermediate in meat accretion. This finding contrasts with results obtained by Clemente et al. (2008) [14], who reported that T had the largest carcass weight but the smallest main cuts yields. In relation to fat deposition, major differences were found between T and R strains. Retinto had significantly greater thickness of total and medium SAT layers than T, while L had intermediate values for TSAT. Torbiscal had a significantly thicker outer SAT layer than the other strains, but Retinto had the greatest IMF, although this finding did not reach statistical significance (*p* = 0.079). These results are similar to those obtained by Ibáñez-Escriche et al. (2016) [41], who reported the lowest values of IMF for the T genotype when compared with R. Former studies such as that of Benito et al. (2000) [10] obtained no significant differences in ham IMF among the three genotypes, and Clemente et al. (2008) [14] found more IMF in the L strain, measured in the tenderloin muscle. Thus, in summary, our study suggested that Torbiscal had the greatest muscle accretion rate, whereas Retinto exhibited the greatest fat deposition ability, at both subcutaneous and intramuscular locations.

In relation to the feeding system effect, in the present study, CF pigs had better prime cuts yields than MF pigs. During the finishing period of the Iberian pig, where there is low muscle growth [16], a high-energy intake leads to an increased subcutaneous and intramuscular fat deposition [18]. In addition, Tejeda et al. (2002) [17] found that the intramuscular fat in biceps femoris was greater in pigs finished in the *montanera* system than in pigs finished with commercial concentrate, due to the greater content in lipids and nitrogen-free extracts of the acorns compared with concentrate feedstuff. In contrast, in the present study there was no significant difference in *L. thoracis* IMF between both feeding systems. However, MF pigs had significantly larger depots of total SAT and of middle and inner SAT layers. When animals are fattened outdoors, another factor potentially affecting fat deposition is the level of exercise, which can increase the energy consumption, thus, reducing fat deposition. In relation to this, Martins et al. (2015) [42] found no significant difference in SAT deposition between free-range and confined Alentejano pigs (a Portuguese genotype very close to the Iberian strains) slaughtered at 100 kg of body weight, and the IMF was significantly smaller in the free-range animals, possibly due to exercise. This study reinforces the idea that the feeding system (MF vs. CF) does not affect IMF in any Iberian strain and in the conditions of this study.

As for the interaction between genotype and feeding system interaction, this effect was significant for the outer and middle SAT layer thicknesses. This quantitative interaction consisted of the fact that, although there were no significant differences for L and R genotypes between the conventional and *montanera* systems, there were significant differences between feeding systems for the T strain, which showed the highest thickness values when pigs were finished in the *montanera* system. As far as we know, this is the first study to evaluate the interaction between Iberian genotypes and feeding system.

### 4.2. Fatty acid Profiles

Fatty acid profiles depend on diet composition and on enzymatic activities, which determine the potential for endogenous synthesis. The Stearoyl-CoA Desaturase enzyme, is a main enzyme involved in fatty acid synthesis which varies between genotypes, and even among different tissues [36]. The results obtained in SAT in the present study clearly indicated an effect of genotype on fatty acid metabolism. The fact that Retinto deposited more oleic acid and MUFA than the other strains was probably associated with its largest expression of the *SCD* gene (coding the Stearoyl-Coenzyme-A Desaturase), which is involved in the de novo synthesis and particularly in the desaturation of fatty acids. The R genotype deposited the greatest MUFA content as well as more IMF (*p* = 0.08) than the other two strains, which was partially in line with the results reported by Poklukar et al. (2020) [38]. The authors established, for local pig breeds different than the Iberian, a direct relationship between a larger IMF and a larger proportion of SFA and MUFA and a smaller proportion of PUFA, although in our study larger IMF was associated only with larger MUFA in R. Similarly, Sellier (1998) [43] and Estévez et al. (2003) [44] stated that leaner Iberian pigs had larger proportions of PUFA in SAT and IMF, and also found negative correlations between PUFA percentage and IMF.

Estévez et al. (2003) [44] found no significant differences for oleic acid, MUFA, SFA, and PUFA among the same three Iberian pig strains at 90 kg of body weight. This discrepancy may be due to the differences in age and body weight between studies. Probably, along the finishing period (well beyond 90 kg), the differences in lipid metabolism among the three strains become increasingly more evident as body weight and fat deposition rise. On the other hand, Juárez et al. (2009) [9] compared these three Iberian strains at an average body weight of 160–180 kg and found no differences in MUFA and PUFA, but the Torbiscal had larger SFA deposition than the other two strains in fat from tenderloin samples, therefore, an effect of the muscle on fatty acid profiles cannot be discarded. Additionally, Muriel et al. (2004) [13], compared four Iberian strains (the three ones analysed here plus Entrepelado), slaughtered at 150 kg of body weight and fed in a *montanera* system, and reported no significant differences in fatty acid deposition among genotypes. Cava et al. (2003) [45] also did not find significant differences among Iberian strains in fatty acid composition, contrary to the results from the present study. A possible reason could be that 150 kg. was a low slaughter weight for the R, T, and Entrepelado strains, that probably were not fully fattened.

When comparing the Iberian pig with other commercial breeds [46,47], the Iberian breed reported significantly lower PUFA deposition. Moreover, the smaller observed proportion of SFA in the Iberian compared with other breeds (Celta) leads to a lower n6/n3 ratio and lower AI and TI indexes, resulting in a healthier fat, and, thus, lowering the risk of coronary heart disease [48]. Therefore, considering the values of SFA, MUFA and PUFA from the three strains of the present study, it can be postulated that Retinto has the healthiest fat.

In our study, as expected, there were important differences in subcutaneous fatty acid composition between feeding systems. Pigs finished in the *montanera* system presented significantly higher percentages of oleic, linoleic, and linolenic acid than those finished with concentrate (53 vs. 50, 11 vs. 10, and 2 vs. 1, respectively), and the latter exhibited larger values for SFA, C16:1, C17:1 and C20:1, thus, MF pigs also had greater values of total MUFA and PUFA. These results are in agreement with those previously reported for intramuscular fat composition [18,49,50] and for subcutaneous fat [49,51,52] in Iberian pigs.

The characteristic fatty acid profile of *montanera*-fed animals is a consequence of the high oleic acid content of acorns, which are consumed in large amounts [20,21]. The latter authors also reported that Iberian pigs fed with acorns had larger proportions of MUFA and PUFA than those fed a concentrate-based diet, matching our results. Additionally, Benítez et al. (2018) [22] concluded that a concentrate diet supplemented with a high concentration of oleic acid (similarly to *montanera* diet) resulted in higher MUFA content and lower SFA content than a conventional diet. In the present study, the n6/n3 index was significantly smaller for acorn-fed animals than for those fed concentrate (7.3 vs. 10.2, respectively), however, these two values were above the health-recommendation proposed by Simopoulos (2004) [53]. In relation to the AI and TI indexes, both were significantly smaller for the MF than for the CF pigs, Therefore, as has been stated before, and according to the general consensus among scientists and industrial stakeholders, the results of the present study support the concepts that fat from Iberian pigs fed acorns in a *montanera* system is healthier than fat from pigs finished with commercial concentrates, and also that, as a counterpart, the former has a greater content of PUFA, which may result in a higher potential predisposition for oxidation.

### 4.3. Gene Expression

Results from this study indicate significant differences in expression for *SCD*, *ME1*, *ACACA*, *FASN*, *ACOX* and *ACLY* genes among Iberian genotypes and feeding systems. The *SCD* (Stearoyl-CoA desaturase) gene, responsible for the biosynthesis of MUFA, mainly C18:1 from the desaturation of stearic acid (C18:0) [54], was previously shown upregulated in Iberian pigs when compared with pure Duroc [22] and with Duroc x Iberian crossbreeds [23]. In the present study, *SCD* gene was over-expressed (two-fold) in the R genotype vs. the T and L genotypes. Very few studies have compared the different Iberian strains for gene expression. Recently, despite not exclusively comparing SCD expression, Villaplana-Velasco et al. (2021) [24] compared Iberian R and T strains and their reciprocal crossbreeds, and found that R strains had a higher content of MUFA and SFA; these differences were explained by the authors as a possible reduction in fatty acid catabolism in R. Estany et al. (2014) [55] found, in the Duroc breed, a single nucleotide polymorphism (AY487830:g.2228T>C), whose g.2228T allele enhances fat desaturation in intramuscular and subcutaneous fat, and this effect was associated with a greater *SCD* mRNA expression in muscle, similar to results obtained by Fernández et al. (2017) [56]. Further studies demonstrated that this polymorphism is segregating in the Duroc breed but is almost fixed (frequency 0.98) in the Iberian pig population [31]. To prove that differential *SCD* expression in the muscle of Iberian strains is not caused by this mutation, the promotor region was sequenced in all our experimental animals, showing homozygosity of allele SCD.g.2228T in all animals from the three strains. The *SCD* gene is not only associated with differences in fatty acid composition but also with total fat content in muscle, as was depicted by Doran et al. (2006) [57], who showed that *SCD* expression was correlated with IMF. In contrast, Estany et al. (2014) [55] found no association between *SCD* gene expression and fat deposition. Therefore, the *SCD* gene might be an interesting candidate biomarker for IMF in swine, at least in the Iberian breed.

Several studies agreed that expression of the *ACACA*, *ME1* and *FASN* genes was greater in the Iberian and Alentejano breeds than in other modern breeds [58,59]. Similarly, Albuquerque et al. (2020) [60] found that *ME1* together with *ACLY* and *SCD* were upregulated in the Alentejano strain when compared with the Bisaro strain. These findings suggest an increased fatty acid metabolism in the pigs from the Mediterranean group. In our study, the ME1 (the malic enzyme codifier gene involved in the de novo synthesis of palmitic acid) was upregulated in Lampiño, which had significantly more C16:0 (palmitic acid), than the other two strains.

*ACOX1* gene encodes the acyl-CoA oxidase, which catalyses the beta-oxidation of very long chain fatty acids, thus, playing an essential role in fatty acid degradation. Although Zuo et al. (2007) [33] found significant differences in this gene expression between Chinese fatty-type pig breeds and western commercial pig breeds, in our study there were no differences among Iberian strains.

The *EGR1* gene encodes a zinc-finger transcription factor implicated in proliferation and differentiation of many cell types and partially responsible for maintaining adequate levels of adipogenesis [32]. Moreover, the *EGR1* has been proposed as a candidate gene for IMF regulation by Wang et al. (2017) [61], however, Muñoz et al. (2018) [31] found no effect of this gene on IMF in Iberian pigs. Similarly, in the present study, EGR1 expression was not affected by genotype or feeding system.

In our study, most of the studied genes related to lipid metabolism were overexpressed in the CF in relation to the MF system except for *SCD* and *EGR1* genes. Similarly, Tejeda et al. (2020) [62] found higher enzymatic activity of ME and *FASN* in pigs finished with conventional feed than those fed in the *montanera* system, which was, according to the authors, due to a greater intake of food in the CF system than in the MF system. In addition, a study carried out by Benítez et al. (2019) [63] in which a high-carbohydrate diet was compared with a high oleic acid diet, resulted in a higher expression of genes related to lipid metabolism in the high-carbohydrate diet than in the high oleic acid diet. Similarly, in our study, in the MF system (high oleic acid diet) most of the studied genes were downregulated. Results could be related with a potential inhibitory effect of the deposited lipids, obtained from the diet, thus, a diet rich in MUFAs may inhibit the expression of lipogenic genes, as has been described for the *SCD* gene which is negatively regulated by oleic and linoleic fatty acids [64]. However, in our study, *SCD* was not affected by feeding system.

To our knowledge, there are no other studies of the interaction between genotype and feeding system on the expression of genes related to lipid metabolism in the Iberian breed. These interactions are significant for the *ACACA* gene, that encodes the acetyl-CoA carboxylase alpha, which is essential in de novo lipogenesis and, therefore, in energy and lipid metabolism [29,65]; for the *FASN* gene involved in the de novo synthesis of palmitic acid; and for the *ACLY* gene that encodes the ATP-citrate lyase (*ACL*) enzyme, which provides energy (from the Krebs cycle) for the synthesis of fatty acids and cholesterol [34]. The *ACACA* and *FASN* genes were upregulated only in R when fed concentrate, but not in the other strains. Finally, the *ACLY* gene was upregulated in the CF system for T and R strains, but differences in expression between CF and MF were considerably larger in the R than in T genotype. Therefore, Retinto was the strain in which the system effect on gene expression was more intense, possibly due to its higher muscle lipogenic nature and higher accumulation of MUFA, with potential repressive effects on lipogenic gene expression in the MF group.

## 5. Conclusions

From the results of this study, it can be concluded that there are important differences between the three Iberian strains in fat and muscle deposition as well as in lipid metabolism. Joint results indicated a higher muscle accretion in T and a higher and healthier fat accumulation in R, supported by composition as well as gene expression data. Retinto animals accumulated more MUFA, and Lampiño had the highest deposition of SFA and PUFA.

Pigs fed concentrates showed higher meat yield and higher expression of genes involved in fatty acid metabolism, while *montanera*-fed animals showed thicker and healthier SAT (higher MUFAs and PUFAs, lower IA and TI indexes) without differences in IMF. The results agreed with the direct deposition of dietary fat in *montanera* pigs and an activation of endogenous lipid synthesis in CF, possibly due to a higher energy or to a lower oleic acid supply.

The reported results may have technical and economic implications as the phenotypic differences observed among strains suggest the possibility of adapting curing procedures to the specific genotypes, and support the potential improvement of the current labeling system by including the strain, for better categorization of the products. Findings also support the relevance of the traditional extensive system for optimizing the sensorial and nutritional quality of Iberian pig products, in addition to the known implications of the *montanera* system on the sustainability and contribution to the preservation of the *dehesa* ecosystem.

## Figures and Tables

**Table 1:** Brief description of genes studied: their functions and products.

Gene	Protein Synthesised	Function
*SCD*	Stearoyl-Coenzyme A Desaturase	De novo synthesis of MUFA, oleic and palmitoleic acid synthesis [27].
*ME1*	Malic Enzyme	Oxidative decarboxylation of L-malate to pyruvate. NADP–NADPH reduction [28].
*ACACA*	Acetyl-CoA Carboxylase α	Carboxylation of acetyl-CoA to malonyl-CoA. β-oxidation of mitochondrial fatty acid [29].
*FASN*	Fatty Acid Synthase	Synthesis of palmitate from acetyl-CoA and malonyl-CoA. Synthesis of long-chain saturated fatty acids [30]. De novo synthesis of palmitic
*EGR1*	Early Growth Response Gene-1	Adipocyte differentiation, myogenesis and adipogenesis [31,32].
*ACOX*	Acyl CoA Oxidase 1	Desaturation of acyl-CoA to 2-trans-enoyl-CoAs. Degradation of long fatty acid chain [33].
*ACLY*	ATP Citrate Lyase	Synthesis of cytosolic acetyl-CoA. Biosynthesis of fatty acids, cholesterol, and acetylcholine Conversion of citrate to oxaloacetate in Krebs cycle [34,35].

**Table 2:** Sequence of the primers used for the selected genes.

Gene	Forward Primer Sequence	Reverse Primer Sequence
*SCD*	TCCCGACGTGGCTTTTTCTTCTC	CTTCACCCCAGCAATACCAG
*ME1*	GCCGGCTTTATCCTCCTCT	TCAAGTTTGGTCTGTATTTTCTGG
*ACACA*	CTGAGAGCTCGTTTTGAAGGAATA	TTTACTAGGTGCAAGCCAGACAT
*FASN*	AGTAAGCCCAAGTACAGCGG	CTCACGGAGGAGAAGATCACG
*EGR1*	GAGGGCAGCGGCGGTAACAG	GGGAAAAGACTCTGCGGTCAGGTG
*ACOX*	TGGCGGGCACGGCTATTCT	TGGCTGGGCAGGTCATTCA
*ACLY*	ATCCGGACCATCGCCATCATC	ATCCCGCCGGTGTTTCCAATCT
*TBP*	GATGGACGTTCGGTTTAGG	AGCAGCACAGTACGAGCAA
*B2M*	TTCACACCGCTCCAGTAG	CCAGATACATAGCAGTTCAGG

**Table 3:** Effect of genotype and feeding system on main cut yields, percentage of intramuscular fat and depth of SAT layers.

Trait	Unit	L	T	R	CF	MF	MSE	Gen.	F.S.	Int.
Ham	%	0.222 ^ab^	0.225 ^a^	0.218 ^b^	0.224	0.218	0.000	0.009	0.001	NS
Foreleg	%	0.157 ^a^	0.165	0.166	0.163	0.162	0.000	0.002	0.534	NS
*L. thoracis*	%	0.025	0.027 ^a^	0.024	0.027	0.024	0.000	<0.0001	<0.0001	NS
Prime Cuts	%	0.404	0.417 ^a^	0.407	0.415	0.404	0.000	0.002	0.001	NS
TSAT	cm	8.76 ^ab^	8.19 ^b^	8.87 ^a^	8.14	9.07	0.857	0.037	0.001	NS
ISAT	cm	2.42 ^ab^	2.21 ^b^	2.43 ^a^	2.14	2.57	0.343	0.097	0.000	NS
MSAT	cm	4.89	4.25 ^a^	5.13	4.59	4.92	0.634	0.000	0.092	*
OSAT	cm	1.45	1.72 ^a^	1.31	1.41	1.57	0.240	<0.0001	0.033	**
IMF	%	3.980	4.140	6.310 *	4.670	4.960	1.796	0.079	0.449	NS

L = Lampiño strain; T = Torbiscal strain; R = Retinto strain; CF = conventionally finished (concentrate diet); MF = *montanera*-finished (free-range system, acorn diet); MSE = mean square error; Gen. = genotype; F.S. = feeding system; Int. = interaction; TSAT = total subcutaneous adipose tissue at 10th rib level; ISAT = inner subcutaneous adipose tissue at 10th rib level; MSAT = middle subcutaneous adipose tissue at 10th rib level; OSAT = outer subcutaneous adipose tissue at 10th rib level; IMF = percentage of intramuscular fat (in *L. thoracis* at the 10th rib level); * = *p*-Value less than 0.10; ** = *p*-Value less than 0.05. Within genotype or feeding system columns, means with different superscript letters in a row differ (*p* < 0.05).

**Table 4:** Effect of genotype and feeding system on fatty acid composition (%) and their ratios.

	Fatty Acid Composition and Ratios								
	Genotype			Feeding System		*p*-Value			
Trait	L	T	R	CF	MF	MSE	Gen.	F.S.	Int
C12:0	0.054	0.052	0.054	0.054	0.053	0.005	0.1507	0.3542	NS
C14:0	1.200	1.136 ^a^	1.196	1.197	1.158	0.079	0.0141	0.0485	NS
C16:0	21.670 ^a^	20.740	20.262	21.688	20.094	0.916	<0.0001	<0.0001	NS
C16:1	1.449	1.224 ^a^	1.435	1.511	1.227	0.211	0.001	<0.0001	NS
C17:0	0.431 ^a^	0.325	0.341	0.425	0.307	0.048	<0.0001	<0.0001	**
C17:1	0.488 ^a^	0.422	0.413	0.516	0.366	0.079	0.006	<0.0001	**
C18:0	11.041 ^a^	10.805 ^ab^	10.490 ^b^	11.542	10.015	0.614	0.085	<0.0001	*
C18:1	49.908 ^c^	51.763 ^b^	52.585 ^a^	49.991	52.846	1.964	<0.0001	<0.0001	NS
C18:2	10.509 ^a^	10.128	9.889	9.875	10.476	0.217	0.000	<0.0001	**
C18:3	1.235	1.161 ^a^	1.228	0.972	1.444	0.017	0.129	<0.0001	NS
C20:0	1.340 ^a^	1.499	1.450	1.501	1.359	0.140	0.0018	0.0001	NS
C20:1	0.674	0.713 ^a^	0.665	0.717	0.651	0.060	0.025	<0.0001	NS
SFA	35.738 ^a^	34.569 ^b^	33.783 ^c^	36.420	32.974	2.281	0.001	<0.0001	NS
MUFA	52.519 ^c^	54.142 ^b^	55.100 ^a^	52.734	55.107	2.202	<0.0001	<0.0001	NS
PUFA	11.744 ^a^	11.289	11.116	10.847	11.919	0.294	0.001	<0.0001	*
AI	0.414 ^a^	0.388	0.380	0.418	0.370	0.025	0.0001	<0.0001	NS
TI	0.964 ^a^	0.918	0.885	1.004	0.841	0.066	0.001	<0.0001	NS
Ratio n6/n3	8.869	9.123	8.332 ^a^	10.237	7.312	0.531	0.003	<0.0001	**

L = Lampiño strain; T = Torbiscal strain; R = Retinto strain; CF = conventionally finished (concentrate diet); MF = *montanera*-finished (free-range system, acorn diet); MSE = mean square error; Gen. = genotype; F.S. = feeding system; Int = interactions; SFA = saturated fatty acids; MUFA = monounsaturated fatty acids; PUFA = polyunsaturated fatty acids; AI = atherogenic index; TI = thrombogenic index; Ratio 6/3 = n6/n3; ratio obtained by dividing omega 6 fatty acid by omega 3 fatty acid contents. Within genotype or feeding system columns, means with different superscript letters in a row differ (*p* < 0.05); * = *p*-Value less than 0.10; ** = *p*-Value less than 0.05

**Table 5:** Effect of genotype and feeding system on muscle expression of genes involved in fatty acid metabolism.

	Relative Gene Expression								
	Genotype			Feeding System			*p*-Value		
Gene	L	T	R	CF	MF	MSE	Gen.	F.S.	Int
*SCD*	0.267	0.268	0.559 ^a^	0.347	0.382	0.027	<0.0001	0.412	NS
*ME1*	0.570 ^a^	0.466 ^b^	0.510 ^ab^	0.558	0.472	0.010	0.008	0.001	NS
*ACACA*	0.395	0.436	0.447	0.463	0.389	0.013	0.335	0.012	**
*FASN*	0.089	0.118	0.337 ^a^	0.243	0.119	0.025	<0.0001	0.002	**
*EGR1*	0.169	0.176	0.260	0.239	0.164	0.040	0.255	0.133	NS
*ACOX*	0.645	0.684	0.650	0.697	0.622	0.010	0.390	0.004	NS
*ACLY*	0.305	0.336	0.430 ^a^	0.422	0.292	0.005	<0.0001	<0.0001	**

L = Lampiño strain; T = Torbiscal strain; R = Retinto strain; CF = conventionally finished (concentrate diet); MF = *montanera*-finished (free-range system, acorn diet); MSE = mean square error; Gen. = genotype; F.S. = feeding system; Int = interactions. Within genotype or feeding system columns, means with different superscript letters in a row differ (*p* < 0.05); ** = *p*-value less than 0.05

## Data Availability

Data is contained within the article or supplementary material and raw data is available on request.

## Supplementary Materials

The following supporting information can be downloaded at: https://www.mdpi.com/article/10.3390/ani13071138/s1, Table S1: Nutritional composition of the concentrate-based diet.
