# Supplementary material for: Differences in Muscle Lipogenic Gene Expression, Carcass Traits and Fat Deposition among Three Iberian Pig Strains Finished in Two Different Feeding Systems
Source: Animals (Basel). 2023 Mar 23;13(7):1138. doi: 10.3390/ani13071138 (PMC10092979; doi:10.3390/ani13071138)
Supplement: Supplementary file 1 [file animals-13-01138-s001.zip › animals-2282748-supplementary.pdf]

Supplementary Table S1. Nutritional composition of the concentrate-based diet.

Parameter	Values
Dry matter	12.116 %
Ash	5.046 %
Crude protein	15.734 %
Crude fat	2.628 %
• Saturated Fatty Acids	• 0.98 %
• Monounsaturated Fatty Acids	• 1.41 %
• Polyunsaturated Fatty Acids	• 0.24 %
Crude fibre	4.920 %
Neutral detergent fibre	14.680 %
Metabolizable energy (swine)	3053.321 kcal/kg
Net energy (swine)	2246.490 kcal/kg
Lysine	0.868 %
Methionine	0.260 %
Calcium	0.868 %
Phosphorus	0.571 %
Sodium	0.185 %
E4.copper	24.840 mg/kg
E1.iron	156.833 mg/kg
E5.manganese	68.324 mg/kg
E6. Zinc	154.520mg/kg
E2. Potassium iodide	0.402 mg/kg
E8. Selenium	0.308 mg/kg
Vitamin A	5 000 000 UI/kg
Vitalmin D3	1 000 000 UI/kg
Vitamin E	31.946 mg/kg
Beta-glucanase	250 000 Gluc/kg
Beta-xylanase	560 000 Xyl/kg
E321. Butylated hydroxytoluene (BHT)	0.144 mg/kg
E320. Butylated hydroxyanisole (BHA)	0.024 mg/kg
E324.ethoxyquin	0.053 mg/kg
E562.sepiolite	0.123 %
